# Postnatal development of depth-dependent collagen density in ovine articular cartilage

**DOI:** 10.1186/1471-213X-10-108

**Published:** 2010-10-22

**Authors:** Mark C van Turnhout, Henk Schipper, Barend van Lagen, Han Zuilhof, Sander Kranenbarg, Johan L van Leeuwen

**Affiliations:** 1 Wageningen University, Department of Animal Sciences, Experimental Zoology Group, PO Box 338, 6700 AH, Wageningen, The Netherlands; 2 Wageningen University, Laboratory for Organic Chemistry, PO Box 8026, 6700 EG, Wageningen, The Netherlands

## Abstract

**Background:**

Articular cartilage (AC) is the layer of tissue that covers the articulating ends of the bones in diarthrodial joints. Adult AC is characterised by a depth-dependent composition and structure of the extracellular matrix that results in depth-dependent mechanical properties, important for the functions of adult AC. Collagen is the most abundant solid component and it affects the mechanical behaviour of AC. The current objective is to quantify the postnatal development of depth-dependent collagen density in sheep (*Ovis aries*) AC between birth and maturity. We use Fourier transform infra-red micro-spectroscopy to investigate collagen density in 48 sheep divided over ten sample points between birth (stillborn) and maturity (72 weeks). In each animal, we investigate six anatomical sites (caudal, distal and rostral locations at the medial and lateral side of the joint) in the distal metacarpus of a fore leg and a hind leg.

**Results:**

Collagen density increases from birth to maturity up to our last sample point (72 weeks). Collagen density increases at the articular surface from 0.23 g/ml ± 0.06 g/ml (mean ± s.d., *n *= 48) at 0 weeks to 0.51 g/ml ± 0.10 g/ml (*n *= 46) at 72 weeks. Maximum collagen density in the deeper cartilage increases from 0.39 g/ml ± 0.08 g/ml (*n *= 48) at 0 weeks to 0.91 g/ml ± 0.13 g/ml (*n *= 46) at 72 weeks. Most collagen density profiles at 0 weeks (85%) show a valley, indicating a minimum, in collagen density near the articular surface. At 72 weeks, only 17% of the collagen density profiles show a valley in collagen density near the articular surface. The fraction of profiles with this valley stabilises at 36 weeks.

**Conclusions:**

Collagen density in articular cartilage increases in postnatal life with depth-dependent variation, and does not stabilize up to 72 weeks, the last sample point in our study. We find strong evidence for a valley in collagen densities near the articular surface that is present in the youngest animals, but that has disappeared in the oldest animals. We discuss that the retardance valley (as seen with polarised light microscopy) in perinatal animals reflects a decrease in collagen density, as well as a decrease in collagen fibril anisotropy.

## Background

Articular cartilage (AC) is the thin layer of soft tissue that covers the articulating ends of the bones in diarthrodial joints. Healthy adult AC is characterised by a depth-dependent composition [1-3] and structure [4-7]. These characteristics result in depth-dependent mechanical properties [8-11] that are important for the functions of adult AC, specifically load distribution and the establishment of a low friction environment [11-13].

AC consists of a number of cells (chondrocytes, ≈ 2% to 5% of the wet volume, [14]) embedded in a porous extracellular matrix (ECM) that is saturated with fluid (≈ 80% wet weight). The ECM consists of collagen and negatively charged proteoglycan molecules. Collagen is the most abundant ECM component (≈ 75% of dry weight, e.g. [3]). Both the predominant orientation in the collagen network and the amount of collagen in the network affect the mechanical behaviour, and thus the functioning, of AC [15-18]. The collagen network remodels between birth and maturity: the adult depth-dependent structure is absent at birth [19-23], collagen type I is replaced by collagen type II [24-26], and collagen densities increase [27-29] with depth-dependent variation [21].

Our knowledge on the depth-dependent development of collagen densities is limited. Most studies that investigate postnatal collagen density measure total collagen content as opposed to depth-dependent collagen density profiles [27-29]. Rieppo et al. [21] measured depth-dependent collagen density profiles in porcine AC in three sample points (4 months, 11 months and 21 months) that did not include perinatal animals. Their results [21] are presented with a 80 μm resolution over the depth of the tissue.

The ECM is produced and maintained by chondrocytes that are affected by their local mechanical environment [30,31]. During postnatal development, AC develops a functional depth-dependent composition and structure. Postnatal collagen density profiles are important for our understanding of postnatal AC development. To unravel the (depth-dependent) mechanobiology of the development of ECM structure and composition, we need better time- and space-resolved collagen density profiles. Information on collagen densities is also essential for the interpretation of optical retardation results from polarised microscopy studies (PLM) [32]. The cited studies into postnatal development of collagen orientation [19-23] all use PLM.

We aim to quantify depth-dependent collagen densities in AC between birth and maturity, and with better spatial resolution than previously reported over the depth of the tissue in a model animal. Recently, we measured postnatal collagen orientation remodelling in a group of 48 sheep (*Ovis aries*) divided in ten age groups between birth and maturity [23]. We use the same animals and anatomical sites for the current study. Second, we aim to assess differences in (the development of) collagen density between different anatomical sites of this single joint surface. In our previous PLM study [23] we found a retardance valley between ≈ 30 μm and ≈ 80 μm from the articular surface for all age categories. Since such a valley can be caused by a minimum in collagen densities, or by a decrease in collagen fibre anisotropy [32], we wish to examine whether a valley in collagen density is present at that location.

We use Fourier transform infrared microspectroscopy (FTIRμS) to measure collagen density in AC. The infra-red absorption at a certain wavenumber $A(\bar{\nu })$ is proportional to the amount of absorbing material present in the light path according to the Bouguer-Lambert-Beer absorption law [33,34]:

(1) $$A(\bar{\nu })=a(\bar{\nu })bc$$

with $a(\bar{\nu })$ the (constant) absorption coefficient at wavenumber $\bar{\nu }$, *b *the optical path length and *c *the concentration of the absorbing material. With a constant thickness (optical path length *b*) of histological sections, equation 1 relates absorption directly to concentrations: $A(\bar{\nu })\sim c$. With FTIRμS an absorption spectrum in the infra-red regime is obtained using a polychromatic light source and Fourier transforms of interferograms, as opposed to measuring absorption at individual wavenumbers with monochromatic light sources.

## Methods

### Animals

The animal experiment was described previously [23]. Briefly, we obtained five female sheep for each of nine sample points from a local sheep farm. Sample points occurred at ages 2, 4, 8, 12, 20, 28, 36, 52 and 72 weeks. An additional four stillborn lambs were used (labelled age = 0 weeks). Animals were kept at the farm with their mother until sacrifice or the age of 12 weeks. Animals that were older than 12 weeks were collected at the farm and housed at the universities laboratory animal facility 'Ossekampen' until sacrifice. The total number of animals at the end of the experiment was 48. The number of animals for the first sample point (0 weeks, stillborn) and the last sample point (72 weeks) was four, and the number of animals for the other sample points was five. The experiment was approved by the Wageningen University Animal Experiments Committee.

### Sample preparation

**Figure 1:**
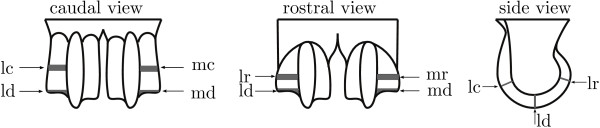
**Sketch of sample sites**. Sketch of the ovine distal metacarpus with the anatomical sampling sites with l - lateral, m - medial, c - caudal, d - distal, and r - rostral.

We used the same tissue blocks to obtain histological slices as those used in our earlier publication [23]. Summarising, the animals' legs were collected immediately following sacrifice and skin and subcutaneous tissue were removed from the metacarpophalangeal joints (figure 1). The joints were carefully opened and we used a dental saw to take the medial and lateral hemispheres from the distal end of each cannon bone. These hemispheres were fixed with formalin and decalcified with EDTA (10% EDTA, pH 7.4) until the hemispheres could be cut with a razor blade. The hemispheres were then divided into a rostral, a distal and a caudal sample (figure 1). Of these, the distal site is expected to be subject to a more static load and the rostral and caudal sites are expected to be subject to a more intermittent load during locomotion [35]. These samples were washed and infiltrated with sucrose (25% sucrose in PBS) overnight, snap frozen in liquid nitrogen and stored at -80°C until further processing, and finally cut to 7 μm thick histological slices with a cryostat (Reichert 2800 N). Histological slices were collected on Potassium Bromide (KBr) disks for FTIRμS analysis.

### FTIRμS system

**Figure 2:**
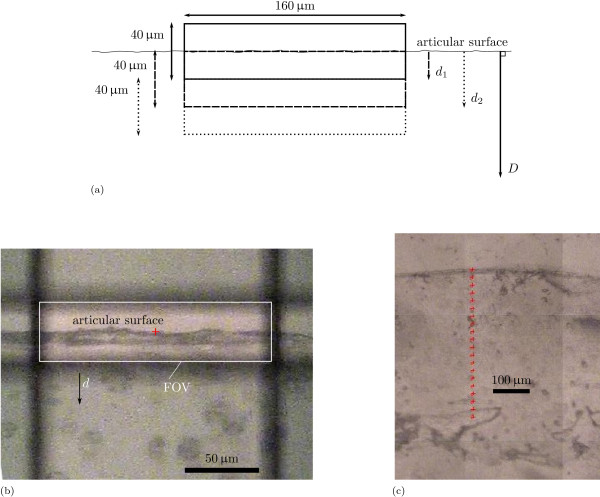
**Example of FTIRμS analysis**. Example of FTIRμS analysis. (a) The rectangular FOV of 160 × 40 μm^2 ^is aligned with the articular surface, and next a linear profile perpendicular to the articular surface with 20 μm intervals is scanned. The depth of the measurement is measured from the articular surface to the centre of the FOV. The first scan is at depth *d*_0 _= 0 μm and only has AC for half of its FOV, the second scan is at *d*_1 _= 20 μm and measures the first 40 μm of the superficial layer, the third scan is at *d*_2 _= 40 μm and measures the FOV for 20 μm ≤ *d *≤ 60 μm, etc. We do not present the results for the first scan at *d*_0_. (b) Example of FOV aligned with the articular surface at *d*_0_. (c) Example of a line scan with 20 μm intervals perpendicular to the articular surface. Crosses show the centre of the (aligned) FOV.

We used a rectangular field of view (FOV) of 160 μm by 40 μm that was aligned with the long side parallel to the articular surface (figure 2). The width of the FOV (160 μm) was chosen to correspond with the width of the FOV in our previous study [23]. The height of the FOV (40 μm) was chosen as a trade off between resolution over the depth of the cartilage, and the necessary assessment time: a FOV with an area of $\frac{1}{x}$ of a given FOV, needs *x*^2 ^more scans to achieve the same signal/noise resolution of the spectra. With this FOV, we scanned a linear profile, along a path perpendicular to the articular surface, with measurements at 20 μm intervals (figure 2c). For each point in each profile, we obtained a single spectrum over the interval 600 cm^-1 ^≤ $\bar{\nu }$ ≤ 4000 cm^-1 ^with the results of 32 scans and with a resolution of 4 cm^-1^.

Two successive FOVs partly overlap to achieve a certain number of scans (> 10) over the depth of the tissue. As a result, we have an a priori smoothing effect for our profiles that uses actual measurements (as opposed to moving averages or linear interpolation). Measurements were performed with a BrukerTensor 27 IR spectrometer, connected to a Bruker Hyperion 2000 IR-microscope (Bruker Optics). This microscope has a liquid nitrogen-cooled MCT-detector - such a detector enables the detection of small amounts of material, ultimately down to monomolecular layers [36]. Both machines are controlled by Bruker's OPUS software. All spectra were baseline corrected with a so-called rubber baseline correction before further processing. This rubber baseline correction consists of finding a convex envelope of the spectrum and subtracting the convex part of the envelope lying below the spectrum from the spectrum [37].

**Figure 3:**
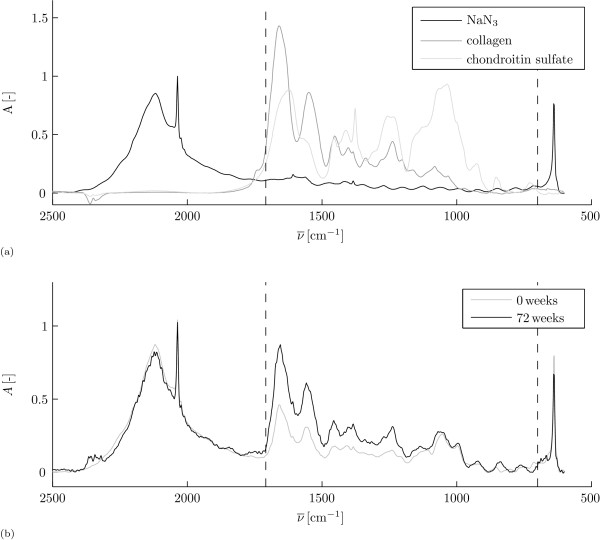
**Example of FTIRμS spectra**. Example of FTIRμS absorption (*A*) spectra. The vertical lines define the parts of the spectra that were used for the quantitative analysis, i.e. 700 cm^-1 ^≤ $\bar{\nu }$ ≤ 1710 cm^-1^. With (a) pure component spectra of the internal standard NaN_3 _(black), collagen (1.35 g/ml, dark gray) and chondroitin sulphate (3.19 g/ml, light gray); and (b) sample spectra from a single FOV at *d *= 100 μm for 0 weeks (gray) and 72 weeks (black).

We used an internal NaN_3 _standard to minimise the effects of variations in the size of the FOV between histological slices [38]. We formed a reference disk of a KBr-NaN_3 _mixture that was placed under the KBr disk that carried the histological slices. The spectrum of NaN_3 _contains a sharp peak at $\bar{\nu }$ = 2036 cm^-1 ^and a sharp peak at $\bar{\nu }$ = 640 cm^-1 ^(figure 3a). We used the peak value of the sharp peak at $\bar{\nu }$ = 2036 cm^-1 ^to normalise all spectra. A single reference disk was used for all measurements and the standard deviation of the peak value in this disk was 3% for 30 measurements over the disk area.

### FTIRμS calibration

Because the ECM consists mostly of collagen type II and glycosaminoglycans (GAGs), we used collagen (bovine tracheal collagen type II, Sigma-Aldrich) and chondroitin-sulphate (bovine tracheal chondroitin sulphate A, Sigma-Aldrich) as standards for pure component spectra. Chondroitin sulphate (or GAG) densities cannot be reliably obtained from FTIRμS spectra [39]. We therefore only quantify collagen densities and we checked whether changes in chondroitin sulphate densities affect our collagen density analysis. We used a least square fitting approach with the data for 700 cm^-1 ^≤ $\bar{\nu }$ ≤ 1710 cm^-1 ^for quantification of collagen content (figure 3b), i.e. we exclude the large NaN_3 _peaks from the fitting procedure. With the pure component spectra we estimated constants *â *and *ĉ *such that the least square difference between the left hand side and right hand side of equation 2 was minimised:

(2) $$\begin{array}{cc} s_{\text{sample}}-s_{{\text{NaN}}_{3}}=\hat{a}s_{\text{col}}+\hat{c} & 700\;{\text{cm}}^{-1} \end{array}\leq \bar{\nu }\leq 1710\;{\text{cm}}^{-1}$$

With *s*_sample _the sample spectrum, *s*_NaN3 _the spectrum of the internal reference, and *s*_col _the spectrum of the collagen standard.

To relate the estimated *â *and *ĉ *to actual collagen densities, we formed and measured a sequence of 36 KBr disks that contained known amounts of the collagen (6 amounts, 0.23 g/ml ≤ *ρ_col _*≤ 1.35 g/ml) and chondroitin sulphate (6 amounts, 0.05 g/ml ≤ *ρ_cs _*≤ 0.32 g/ml) standards. We estimated constants *â *and *ĉ *with equation 2 for this sequence. We applied linear regression to find the constants *b*_1 _and *b*_2 _for the relation between the density of the collagen standard *ρ*_col _and chondroitin sulphate standard *ρ*_cs_, and the estimated constant *â*:

(3) $$\begin{array}{cc} \rho_{\text{col}}=b_{1}\hat{a}+b_{2}, & \rho_{\text{cs}}=b_{3}\hat{a}+b_{4} \end{array}$$

The linear relationship between *ρ*_col _and the estimated *â *(equation 3) is described with equation 4:

(4) $$\begin{array}{cc} p_{\text{col}}=1.45\hat{a}+1.24\cdot 10^{-2}, & r^{2}=0.98 \end{array}$$

In equation 4, zero is included in the 95% confidence interval (-2.92·10^-2 ^≤ *b*_2 _≤ 5.41·10^-2^) for the intercept *b*_2 _= 1.24·10^-2^. The relationship between *ρ*_cs _and the estimated *â *(equation 3) is described by equation 5:

(5) $$\begin{array}{cc} p_{\text{cs}}=1.64\cdot 10^{-2}\hat{a}+1.79\cdot 10^{-1}, & r^{2}=2.2\cdot 10^{-3} \end{array}$$

In equation 5, zero is included in the 95% confidence interval (-1.06·10^-1 ^≤ *b*_1 _≤ 1.39·10^-1^) for the slope *b*_3 _= 1.64·10^-2 ^and the correlation between *ρ*_cs _and *â *is near zero (*r*^2 ^= 2.2·10^-3^). Thus, the analysis of collagen densities is not affected by the amounts of GAG. Because of the near zero intercept for the linear relationship between *ρ*_col _and *â *(*b*_4 _= 1.24·10^-2^, equation 4), we quantified collagen densities with equation 6:

(6) $$p_{\text{col}}=1.45\hat{a}$$

With *â *estimated with equation 2.

To obtain mean depth-dependent profiles between samples, we started at the articular surface (*d*_1 _in figure 2a) and took the mean of the measurements at *d*_1 _of the samples in the pool. We then moved one measurement point towards the calcified tissue and repeated this with *d*_2 _in figure 2a, etc. Note that because of differences in cartilage thickness, the number of samples that we can analyse decreases once we are at a depth larger than *D *for the shortest dataset in the sample pool. We used the exponential fit for cartilage thickness that we found in our previous study [23, equation 2] to show age- and depth-dependent results:

(7) $$D_{f}(t)=618(0.52+{\text{e}}^{-0.11t})$$

with *D_f _*the cartilage thickness in μm and *t *the age in weeks.

### Statistical analysis

Data were analysed with generalized linear mixed models because some of the variables analysed are not normally distributed. Also, measurements on the same animal and position within an animal are dependent. This excludes conventional analyses such as analysis of variance or regression that are intended for normally distributed and independent data. We therefore used the penalized quasi-likelihood methodology described by Schall [40], Breslow & Clayton [41] and Engel & Keen [42]. Calculations were performed with GenStat [43]. The models comprised random effects with associated components of variance, that allowed for dependence between observations of the same animals and the same anatomical sites. Thus, we used a nested structure within animal for hind leg/fore leg, lateral/medial and caudal/distal/rostral sites. In particular, this allowed for additional dependence within animals between duplicate observations on the same site. We are interested in the development of differences between the different anatomical sites with age. Therefore, fixed effects (systematic effects) comprised main effects and all second order interactions for factors age, hind leg/fore leg, lateral/medial site, and caudal/distal/rostral site in the initial models.

Models were fitted separately to three response variables: collagen density at the surface *ρ*_s_, maximum collagen density *ρ*_max_, and the presence of a collagen density valley near the articular surface *ν*. For the variable *ρ*_max _we used an identity link and normal distribution. For the variable *ρ*_max_, we used a log link and gamma variance function, with a multiplicative dispersion parameter. For *v *we obtained the position of the minimum collagen density in the first 5 samples points over the depth. We then scored each collagen density profile with 0 (the minimum is found in the first sample point, i.e. does not results in a valley) or 1 (the minimum occurs after the first sample point and results in a valley). We tested these scores with a logit link (logit *q *= log *q*/(1 - *q*)) and binomial variance function. For each model, random effects on the link scale were assumed to follow normal distributions. Tests were based on an approximate *F *-test [44] applied to the adjusted dependent variate from the last iteration step of the iterative re-weighted restricted maximum likelihood algorithm [42] that we used. The link functions provide the relationship between the linear predictor and the mean of the distribution function and the chosen link and variance functions were needed to achieve satisfactory (normally distributed) residuals for the models. Non-significant (*p *> 0.05) higher order fixed factor interactions were dropped from the initial models.

We used the following symbols in the models: *μ*: intercept; *A_j _*, *j *= 0, 2, 4, 8, 12, 20, 28, 36, 52, 72: age in weeks; *B_k_*, *k *= 1, 2: fixed factor hind leg/fore leg; *C_l_*, *l *= 1, 2: fixed factor lateral site/medial site; *D_m_*, *m *= 1, 2, 3: fixed factor caudal site/distal site/rostral site; *L_i_*: random factor individual animal; and (*LB*)*_ik_*, (*LBC*)*_ikl _*and (*LBCD*)*_iklm _*nested random factors within animal. The final model that we fitted for each covariate, is presented in the results section. We aimed for analysis of two samples for each of six sites (figure 1), for each of two legs, for each of 48 animals, i.e. 1152 samples. Due to the loss of two fore legs and a few missing values, the total number of samples was 1132. In the text, we quantify significant differences as mean ± standard error as predicted by the model. In the figures and text, we use raw means and associated standard deviations, and not model predictions, to present the results.

## Results

**Figure 4:**
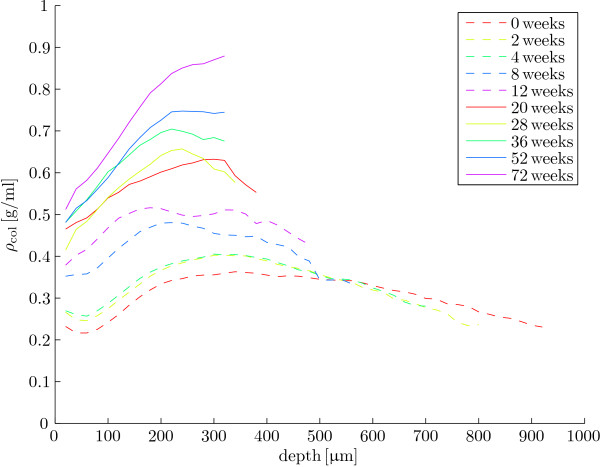
**Collagen density results per age**. Mean collagen density as a function of cartilage depth for the ten ages.

**Figure 5:**
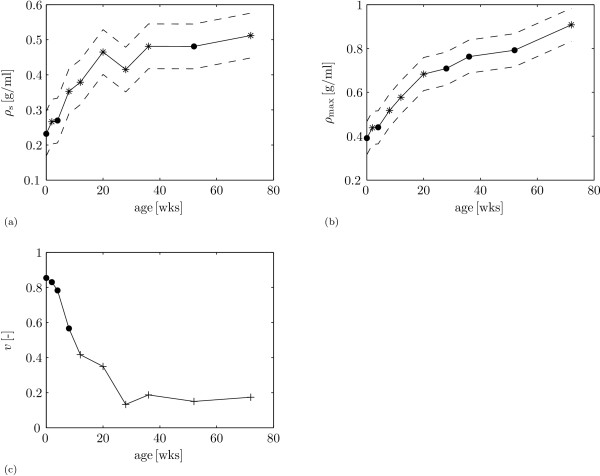
**Statistical results for collagen densities**. Statistical results for collagen density profiles. With (a) mean collagen density at the surface *ρ_s _*(solid) ± standard deviation (dashed) as a function of age. Stars mark values that are significantly different from the value at the previous age; (b) mean maximum collagen density *ρ*_max _(solid) ± standard deviation (dashed) as a function of age. Stars mark values that are significantly different from the value at the previous age; and (c) mean scores for the presence of the collagen density valley *ν *as a function of age. Crosses mark values that are significantly different from the value at 0 weeks.

We show examples of sample spectra for 0 weeks and 72 weeks (figure 3b). Analysis of all spectra with equations 2 and 6 yields the averaged collagen densities per age group (figure 4) and the statistical results (figure 5).

The final model for the collagen density at the surface *ρ*_s _is

(8) $$y_{ijklm}=\mu +A_{j}+B_{k}+C_{l}+D_{m}+{\left(AD\right)}_{jm}+L_{i}+{\left(LB\right)}_{ik}+{\left(LBC\right)}_{ikl}+{\left(LBCD\right)}_{iklm}$$

with *y*_*ijklm *_the predictor. There is a main effect for the factor age for *ρ*_s _(table 1: *A*_*j *_, *p *< 0.001). In general, *ρ*_s _increases with age, up to the last sample point: from 0.23 g/ml ± 0.06 g/ml (mean ± s.d., *n *= 48) at 0 weeks to 0.51 g/ml ± 0.10 g/ml (mean ± s.d., *n *= 46) at 72 weeks (figure 5a). An exception is the measurement at 28 weeks that is lower than the value at 20 weeks and 36 weeks. The interaction of the fixed factor age with the fixed factor caudal site/distal site/rostral site (table 1: (*AD*)*_jm_*, *p *= 0.026) shows significant effects at 4 weeks and 72 weeks, where *ρ*_s _is lower for the rostral site than for the distal site and caudal sites (table 2).

**Table 1:** Results for the statistical model for the collagen density at the surface

fixed term	***A***_***j***_	*B_k_*	*C_l_*	*D_m_*	*(AD)_jm_*
*p*-value	< 0.001	0.207	0.212	0.966	0.026

Results for the statistical model for the collagen density at the surface *ρ*_s _with log-link and gamma variance function. Letters for the fixed terms represent: *A_j _*- age, *B_k _*- fore/hind, *C_l _*- lateral/medial and *D_m _*- caudal/distal/rostral. Combinations represent interactions between these fixed factors.

**Table 2:** Mean collagen density at the surface for the caudal site, distal site, and rostral site

age [weeks]	0	2	4	8	12	20	28	36	52	72
*ρ*_s _[g/ml] caudal	0.22	0.27	0.26*	0.36	0.37	0.45	0.43	0.48	0.49	0.53*
*ρ*_s _[g/ml] distal	0.25	0.27	0.26*	0.34	0.38	0.46	0.40	0.48	0.47	0.54*
*ρ*_s _[g/ml] rostral	0.23	0.26	0.30	0.36	0.39	0.48	0.42	0.47	0.48	0.46

Mean collagen density at the surface *ρ*_s _[g/ml] per age for caudal site, distal site and rostral site from the statistical model. The associated standard error is 0.0155 [g/ml]. Stars mark values that are significantly different from the value for the rostral site at that age.

The final model for the maximum collagen density *ρ*_max _has no fixed factor interactions and is

(9) $$\eta =\mu +A_{j}+B_{k}+C_{l}+D_{m}+L_{i}+{\left(LB\right)}_{ik}+{\left(LBC\right)}_{ikl}+{\left(LBCD\right)}_{iklm}$$

where the conditional expectation of *y*_*ijklm *_for given *η *is logit^-1^(*η*), the inverse of the link function. There is a main effect for the factor age for *ρ*_max _(table 3: *A*_*j *_, *p *< 0.001). *ρ*_max _increases monotonically with age, up to the last sample point: from 0.39 g/ml ± 0.08 g/ml (mean ± s.d., *n *= 48) at 0 weeks to 0.91 g/ml ± 0.13 g/ml (mean ± s.d., *n *= 46) at 72 weeks (figure 5b). There is a significant effect for the factor lateral site/medial site for *ρ*_max _(table 3: *C_l_*, *p *= 0.006): maximum collagen density is 2.72% ± 1.00% (mean ± s.e.) higher at the medial site than at the lateral site. The final model for the presence of a collagen density valley *ν *has no fixed factor interactions and is therefore equal to equation 9. There is only a significant effect for the factor age for *ν *(table 4: *A*, *p *= 0.007): *ν *decreases between birth and maturity (figure 5c). There are no significant differences between successive ages (figure 5c). However, a significant decrease in the score for *ν *occurs between 0 weeks and 12 weeks. The score *ν *at 72 weeks does differ significantly from *ν *at 12 weeks. The mean score at 0 weeks is 0.85, i.e. at this age ≈ 85% (41 out of 48) of the measurements shows a valley in the first 5 sample points over the depth (figure 5c). The mean score at 12 weeks is 0.41 (25 out of 60), and the mean score at 72 weeks is 0.17 (8 out of 46).

**Table 3:** Results for the statistical model for the maximum collagen density

fixed term	*A_j_*	*B_k_*	*C_l_*	*D_m_*
*p*-value	< 0.001	0.169	0.006	0.422

Results for the statistical model for the maximum collagen density *ρ*_max _with log-link and gamma variance function. Letters for the fixed terms represent: *A_j _*- age, *B_k _*- fore/hind, *C_l _*- lateral/medial and *D_m _*- caudal/distal/rostral.

**Table 4:** Results for the statistical model for the presence of a collagen density valley

fixed term	*A_j_*	*B_k_*	*C_l_*	*D_m_*
*p*-value	0.007	0.873	0.480	0.063

Results for the statistical model for the presence of a collagen density valley *ν *with logit-link and binomial variance function. Letters for the fixed terms represent: *A_j _*- age, *B_k _*- fore/hind, *C_l _*- lateral/medial and *D_m _*- caudal/distal/rostral.

## Discussion

As expected [21,27-29], we find that collagen content increases with age between birth and maturity: both *ρ_s _*(figure 5a) and *ρ*_max _(figure 5b) show a positive correlation with age. Contrary to the cartilage thickness and collagen orientation parameters that we measured in the same animals [23], collagen density does not appear to stabilise between 36 weeks and 72 weeks. It thus appears that the potential for collagen remodelling is different between collagen orientation and collagen density. The potential for collagen reorientation appears to be correlated to changes in cartilage thickness [23], whereas collagen density in the current study still increases after cartilage thickness has stabilised in these animals (36 weeks, [23]). Both an increase in the number of collagen fibrils and an increase in collagen fibril thickness results in increased collagen densities. Neither polarised light microscopy, which we used in our previous study [23], nor FTIRμS in the current study is capable of measuring collagen fibril thickness. Additional measurements, e.g. with electron microscopy, will be necessary to elucidate to what degree collagen fibril thickness contributes to the observed collagen density.

**Figure 6:**
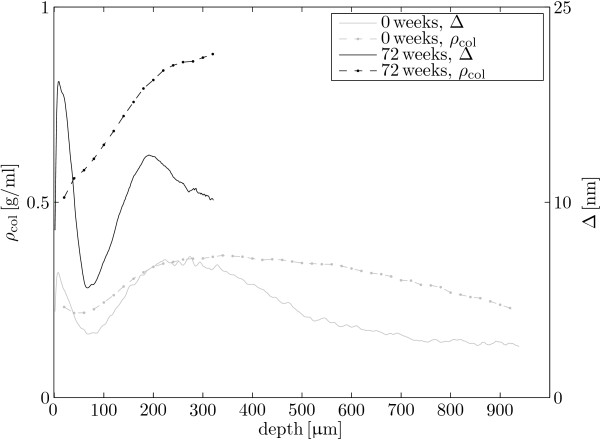
**Interpretation of retardance valley near the articular surface**. Mean collagen densities (*ρ*_col_, dashed) for 0 weeks (gray) and 72 weeks (black) together with mean retardance results (Δ, solid) from our previous study [23]. The valley in collagen density can partly explain the retardation valley in the youngest animals, but not in the oldest animals.

In the current study, we investigated the possible presence of a valley in collagen density near the articular surface, prompted by the presence of a retardance valley at that location in our polarised light microscopy study on the same animals [23]. The data on porcine AC by Rieppo et al. also shows some evidence for a valley in collagen density near the articular surface for the youngest age group, but not for the older age groups [21, figure 2a]. Similarly, we find strong evidence for a valley in collagen density in the youngest animals that disappears with increasing age (table 4, figure 5c). This means that the retardance valley near the articular surface that is present in all age categories [23], must be interpreted differently for the youngest than for the oldest animals (figure 6). The retardance patterns measure primarily a combination of collagen densities and collagen fibril anisotropy [23,32] and the collagen density results (figure 6) show that the retardance valley reflects a decrease in collagen fibril anisotropy, and not a decrease in collagen density, in the adult animals. A decrease in collagen fibril anisotropy is the traditional interpretation of decreased retardance near the articular surface [4,6,45,46]. Our current results suggest that interpretation of retardance valleys near the articular surface in immature AC is less straightforward: the retardance valley in perinatal animals also reflects a decrease in collagen density (figure 6).

Hunziker et al. [47] showed that AC grows appositionally. The superficial zone supplies the stem cells for AC growth. Daughter cells that are displaced horizontally, remain confined to the superficial zone and replenish the stem-cell pool and affect lateral growth. Daughter cells that move vertically downwards form a zone with a rapidly dividing and proliferating pool of cells for rapid clonal expansion. This zone affects longitudinal growth and is located at the transitional and upper deep layer of AC [47]. The location (distance from the articular surface) of the collagen density valley in our study appears to coincide with the zone of rapidly dividing daughter cells in the study by Hunziker et al. [47]. Hunziker et al. further showed that the proliferation activity of this pool of cells decreased with age and had ceased when AC thickness stabilised. The valley in collagen density that we observe in our study also gradually disappears with age, and also stabilises when cartilage thickness stabilises (36 weeks, figure 5c). These similarities in the spatial and temporal patterns of cell proliferation and the presence of a collagen density valley, suggest a relationship between the cell activity and collagen production in this zone. Dedicated investigations will be required to show whether or not such a relationship exists.

FTIRμS is a technique that has gained popularity for the investigation of the collagen network of AC in the last decade [48]. FTIRμS was first applied to biological tissues when it became possible to use a microscope in the light path [49]. The first applications of FTIRμS for AC were investigated at the start of the current century [50,51]. Biochemical analysis of hydroxyproline content is an alternative and well-established technique to quantify collagen density in AC. However, this technique has a limited spatial resolution and is used to measure total collagen content as opposed to depth-dependent profiles, e.g. [27-29]. The main benefit of FTIRμS is the high spatial resolution that can be achieved [39]. With histological slices as in our study, FTIRμS is a relatively easy and fast technique to obtain depth-dependent collagen density profiles in AC. Apart from the overlapping spectra of the AC components (figure 3a), quantitative FTIRμS is challenging because small changes in peak shape and position may occur as a result of the local composition or structure of the proteins [39]. The baseline correction finally, lacks a theoretical or physical background and its implementation is to a certain degree arbitrary. In our experience, the choice of baseline correction hardly influences the quantitative results, as long as the same baseline correction is applied to all samples and calibration sequences.

We treated the samples with sucrose and this hinders analysis of GAG densities because the FTIRμS signal of sucrose interferes with the signal of the sugar groups in the GAGs. When we attempted GAG quantification on samples that were not treated with sucrose, we found similar to Rieppo et al. [39] that GAG densities can not be reliably obtained from FTIRμS spectra. This is probably because collagen is much more abundant in AC than the GAGs [3], and because the absorption for collagen is higher than for chondroitin sulphate for equal amounts of pure components (figure 3a).

Several approaches have been used for quantitative analysis of AC FTIRμS spectra, e.g. integrated peak areas [50], (partial) least square fits [52], Euclidean distance analysis [51] and deconvolution approaches [39]. Analysis with integrated peak areas is the most straightforward, and with our calibration sequence we estimated an error of 5% for collagen densities for a 4:1 ratio of collagen to chondroitin sulphate [1,3]. However, since the development of the ratio of collagen to chondroitin sulphate is unknown, we selected a different approach to analyse our spectra because we could show that chondroitin sulphate density does not affect the parameter *â *(*r*^2 ^= 2.2 · 10^-3^, equation 5).

The spatial and temporal collagen density profiles in the current study are similar to those in a previous FTIRμS study into postnatal AC development by Rieppo et al. [21]. Rieppo et al. [21] looked at domestic pig AC from the femoral groove at 4 months, 11 months and 21 months of age. As in our study, they found that collagen density shows a maximum between the superficial and deep zone in the youngest age group, and that collagen density increases monotonically between the superficial and the deep zone for the oldest animals [21, figure 4a]. Also, the order of magnitude of collagen density in the adult animals in our study is in line with previous reports on total AC density, e.g. ≈ 1.4 g/ml for bovine [2] and human AC [17].

In our previous study on collagen orientation [23], we observed that the caudal site developed differently from the distal and rostral sites. We can not show such a difference in the current study. We do find significant but small (2.7%) differences in *ρ*_max _between lateral sites and medial sites (medial sites higher). In our previous study [23], we found an effect for the lateral sites and medial sites for the collagen orientation pattern (superficial zone thicker at lateral site). Whether or not these effects are related cannot be resolved from these studies. Dedicated finite element models can be an aid for a functional analysis of these effects, e.g. [10,18]. In our previous study [23] we found that differences during development had disappeared in the last sample point (72 weeks). In that study, we explained the lack of differences in the mature animals by the near congruent joint that we investigated. Our current results support that explanation: we observe very little differences in collagen density over this near congruent joint surface. It thus appears that the expected different loading regimes for the different sites (more static at the distal site, intermittent at the caudal and rostral sites) have little influence in the joint and animal that we investigated.

Finite element models can also assist in a functional analysis of the depth-dependent collagen density development in postnatal life, e.g. [10,29,53]. We performed such a functional analysis [18] with our earlier data on collagen orientation remodelling [23]. In that functional analysis [18] we found a marked increase in AC stiffness near the bone, but not at the articular surface. Thus, the effect of postnatal collagen reorientation is the (further) development of depth-dependent mechanical properties in AC. These depth-dependent mechanical properties of AC are thought to be important for the adult functions of AC [8,12,54,55]. In the current study, collagen density increases most in the deep cartilage. Because cartilage stiffness correlates positively with collagen density [16,56] we hypothesise that this depth-dependent distribution of collagen density also contributes to the development of a depth-dependent gradient in mechanical properties of AC in postnatal life.

With this paper, we complement our earlier data on postnatal collagen reorientation in the same animals [23]. The combination of the two data sets provides better tools for functional analysis of the role of the collagen fibre network during development, e.g. by composition based finite element models [10,18,53]. Also, the combination of retardance results and collagen density results (figure 6) enabled us to further illustrate the peculiar nature of the transitional zone in the perinatal animals [20,23]. We thus contribute to a better understanding of the mechanobiology of articular cartilage development. With additional information on the development of GAG concentrations and fixed charge densities in the AC, it becomes possible to estimate the mechanical environment that drives the depth-dependent AC development in general, and depth-dependent collagen remodelling in particular.

## Conclusions

Collagen densities in articular cartilage increase in postnatal life with depth-dependent variation: the increase in collagen density at the articular surface is smaller than the increase in maximum collagen density in the deep cartilage. Collagen density does not stabilise by 72 weeks, the last sample point in this study. Because cartilage stiffness correlates positively with collagen density [16,56], we predict that the depth-dependent pattern of collagen density remodelling contributes to the functional depth-dependent gradient in the mechanical properties of AC.

We find strong evidence for a valley in collagen densities near the articular surface (*d *< 100 μm) that is present in the youngest animals, but that has disappeared in the oldest animals. A valley in retardance near the articular surface is traditionally interpreted as the result of a decrease in collagen anisotropy. Our current results show that the retardance valley in perinatal animals also reflects a decrease in collagen density.

## Authors' contributions

MvT carried out the design of the study and its coordination, the data acquisition (FTIRμS-measurements), processing, (statistical) analysis and interpretation, and drafting the manuscript. HS carried out the acquisition of data (sample preparation & histology), and participated in data analysis and interpretation and drafting the manuscript. BvL assisted with the data acquisition and participated in the interpretation of data and critical revisions of the manuscript. HZ participated in the interpretation of data and critical revisions of the manuscript. SK and JvL participated in the design of the study and its coordination, data analysis and interpretation and critical revisions of the manuscript. All authors read and approved the final manuscript.
